# Cytoplasmic Dynein Promotes HIV-1 Uncoating

**DOI:** 10.3390/v6114195

**Published:** 2014-11-04

**Authors:** Paulina Pawlica, Lionel Berthoux

**Affiliations:** Laboratory of Retrovirology, Department of Medical Biology and BioMed Research Center, Université du Québec à Trois-Rivières, 3351 Boulevard des Forges, Trois-Rivières, QC G9A 5H7, Canada; E-Mail: paulina.pawlica@uqtr.ca

**Keywords:** HIV-1, uncoating, capsid, microtubules, dynein

## Abstract

Retroviral capsid (CA) cores undergo uncoating during their retrograde transport (toward the nucleus), and/or after reaching the nuclear membrane. However, whether HIV-1 CA core uncoating is dependent upon its transport is not understood. There is some evidence that HIV-1 cores retrograde transport involves cytoplasmic dynein complexes translocating on microtubules. Here we investigate the role of dynein-dependent transport in HIV-1 uncoating. To interfere with dynein function, we depleted dynein heavy chain (DHC) using RNA interference, and we over-expressed p50/dynamitin. In immunofluorescence microscopy experiments, DHC depletion caused an accumulation of CA foci in HIV-1 infected cells. Using a biochemical assay to monitor HIV-1 CA core disassembly in infected cells, we observed an increase in amounts of intact (pelletable) CA cores upon DHC depletion or p50 over-expression. Results from these two complementary assays suggest that inhibiting dynein-mediated transport interferes with HIV-1 uncoating in infected cells, indicating the existence of a functional link between HIV-1 transport and uncoating.

## 1. Introduction

The mature capsid (CA) core of human immunodeficiency virus 1 (HIV-1) is a ~60 nm × 120 nm [1] cone-shaped protein lattice, encasing viral genomic RNA, enzymes (integrase and reverse transcriptase) and other viral proteins (reviewed in [2]). It is composed of ~1000 CA monomers [3] arranged in ~170 hexamers and 12 pentamers through interactions involving their N-terminal domains [4], whereas the C-terminal domains are important for homodimerization that connects the rings into a lattice [5]. Following receptor-mediated entry into the cytoplasm, HIV-1 undergoes tightly regulated processes that prepare the virus for nuclear import and integration of its genetic material into host DNA. The nucleoprotein complex formed before completing reverse transcription (RT) is usually referred to as the reverse transcription complex (RTC), which is later transformed into the pre-integration complex. RT is thought to be initiated within 30 min post virus entry [6] by a still unknown mechanism. Before, during or after RT, the CA core must be disassembled in a process called “uncoating”. The precise timing and location of uncoating remain unclear; some reports propose that it occurs in the cytoplasm [7,8], while others suggest that uncoating takes place at the nuclear pore [9,10,11]. It is possible that a small fraction of CA proteins remain associated with pre-integration complexes and are transported to the nucleus [12]. These hypotheses are not mutually exclusive, considering that uncoating could take place through several sequential steps rather than through a rapid single-step process [13]. Additionally, recent lines of evidence indicate that uncoating is linked with RT [8,14,15] and that RT may even trigger uncoating [8,16]. However, RT initiation itself may depend on prior destabilization or increased permeability of the CA core after entry into the cytoplasm [17]. Alternatively, uncoating could be triggered by an as yet unknown cellular factor [18].

Numerous studies indicate that CA is the main viral determinant for uncoating. Mutations resulting in either hypostable or hyperstable CA cores almost completely abolish viral infectivity despite normal virion protein content [19]. Additionally, the retroviral restriction factors TRIM5α and TRIMCyp inhibit viral infection [20,21] through binding to and destabilization of incoming CA cores, in essence causing “premature uncoating” [22]. Furthermore, the HIV-1 small-molecule inhibitor PF-3450074 (PF74) inhibits HIV-1 infectivity specifically by destabilizing the CA core [23]. Uncoating is also likely dependent on cellular factors, but these remain mostly elusive [13]. A few cellular partners that interact with the HIV-1 CA core were characterized and some were proposed to modulate uncoating, such as cyclophilin A (CypA) [24,25], nucleoprotein RanBP2/Nup358 [10], transportin 3 (TNPO3) [25], cleavage and polyadenylation specific factor 6 (CPSF6) [26] and PDZ Domain-containing 8 [27].

Similarly to other viruses (reviewed in [28]), HIV-1 hijacks the cell’s cytoskeleton and associated molecular motors for its trafficking towards the nucleus (reviewed in [29]). The microtubule network and microtubule-associated dynein motor complexes were proposed to transport HIV-1 RTCs toward the nucleus [7]. Specifically, HIV-1 translocation on microtubules was evidenced by live microscopy, and was inhibited by micro-injection of anti-dynein antibodies [7]. Recently, it was reported that HIV-1 induces the formation of and co-localizes with a stable sub-population of microtubules whose disruption abolishes infectivity [30]. Thus, the microtubule network seems to be important for HIV-1 trafficking and infectivity.

In this study, we hypothesized that uncoating of the HIV-1 CA core and its trafficking towards the nucleus were linked mechanisms. Our results suggest a role for dynein motor complexes in HIV-1 uncoating.

## 2. Results

### 2.1. Disruption of the Dynein Motor Complex Causes an Accumulation of CA Foci in Infected Cells

**Figure 1:**
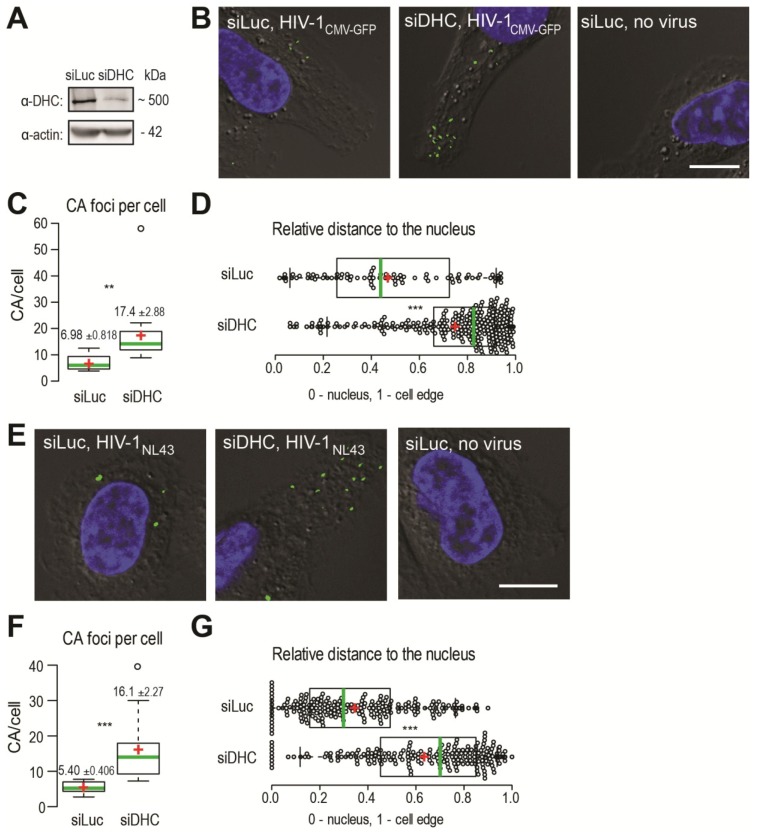
Dynein heavy chain (DHC) depletion causes the accumulation of HIV-1 CA cores in infected cells and alters their subcellular distribution. (**A**) HeLa cells were transfected with siRNAs targeting either dynein heavy chain (siDHC) or luciferase (siLuc) as a control, and DHC expression was analyzed 2 days later by Western blotting; (**B**) Immunofluorescence microscopy observations of CA foci following infection with VSV G-pseudotyped HIV-1. HeLa cells were transfected with the indicated siRNAs and 72 h later were infected or not with VSV G-pseudotyped HIV-1_CMV-GFP_ in the presence of MG132. At 4 h p.i., supernatants were replaced with virus-free medium. Cells were fixed 6 h p.i. and stained to detect CA (green) or DNA (blue). The outline of cells was revealed by low-exposure bright-field microscopy. Representative images are shown. The white bar represents 10 μm; (**C**) Box plots showing total amounts of CA foci per cell. The total numbers of CA foci and nuclei in 10 randomly chosen fields were counted and the CA foci/nuclei ratios were calculated. 375 and 1047 CA foci were counted for siLuc and siDHC, respectively. Center green lines show the medians; box limits indicate the 25th and 75th percentiles as determined by R software; whiskers extend 1.5 times the interquartile range from the 25th and 75th percentiles; and red crosses represent sample means. ** indicates *p* ≤ 0.001 in a Student *t*-test analysis; (**D**) Box plots showing the relative localization of CA foci in 10 randomly chosen cells, calculated using the formula x/(x + y) where x is the shortest distance to the nucleus edge and y is the shortest distance to the cell’s edge in the two-dimensional cellular cross-section analyzed. Data points are plotted as open circles; center green lines show the medians; box limits indicate the 25th and 75th percentiles as determined by R software ; whiskers extend 1.5 times the interquartile range from the 25th and 75th percentiles; and red crosses represent sample means. *** indicates *p* ≤ 0.0001 in a Student *t*-test analysis; (**E**) Immunofluorescence microscopy observation of CA foci following infection with HIV‑1 bearing an autologous envelope. MAGI cells were transfected with siDHC or siLuc prior to infection, and 72 h later were infected with HIV-1_NL43_ in the presence of MG132. Cells were fixed 2 h later and stained as in (**B**); The white bars represent 10 μm; (**F**) The total amounts of CA foci per cell were calculated as in (**C**); At least 250 CA foci were counted for each condition (*** indicates *p* ≤ 0.0001); (**G**) Relative cellular distribution of CA foci, analyzed as in (**D**) (*** indicates *p* ≤ 0.0001).

We used immunofluorescence (IF) microscopy to analyze the presence of CA foci, visualized using a CA monoclonal antibody, in human HeLa cells infected with HIV‑1_CMV-GFP_ pseudotyped with the glycoprotein of the vesicular stomatitis virus (VSV G) or in human MAGI cells infected with HIV-1_NL43_. Such CA foci detected in acute infection conditions are thought to be individual viruses that are not or only partially uncoated [7,8,9,31]. HeLa cells transfected with siRNAs targeting either the dynein heavy chain (DHC) mRNA [32,33] or the non-relevant luciferase (Luc) mRNA [33] were infected with HIV‑1_CMV‑GFP_ for 2 h, at which point supernatants were removed and cells were incubated for an additional 4 h. This timing was previously determined to allow for the efficient detection of cytoplasmic, unenveloped HIV-1 CA cores [34]. DHC depletion was efficient as assessed by Western blotting, since transfection of DHC siRNA led to a reduction of ~85.4% ± 6.7% (*n* = 10) in protein levels (Figure 1A), which is consistent with previous observations that a large proportion of cells showed altered dynein-dependent transport in these conditions as seen by LAMP-1 staining [33]. Upon staining infected cells using an anti-CA antibody, we detected distinct foci that were absent from mock-infected cells, as expected (Figure 1B). We analyzed the median slice from a Z-stack for each field, which removed the cytoplasm present on top of or underneath the nucleus from the analysis. DHC depletion caused an increase in the amounts of CA foci. Specifically, the average number of CA foci per cell increased ~2.5-fold following DHC depletion, from 6.98 ± 0.82 in control cells (*n* = 71 cells analyzed) to 17.4 ± 2.9 in DHC-depleted cells (*n* = 57) (*p* = 0.0038) (Figure 1C). Moreover, CA foci tended to accumulate in the cell periphery, as evidenced by measuring relative distances to the nucleus. The median relative distance to the nucleus changed significantly (*p* < 0.0001) from 0.44 (95% CI, 0.39–0.52) for the Luc siRNA-transfected control cells (*n* = 10) to 0.83 (95% CI, 0.79–0.84) for cells (*n* = 16) transfected with DHC siRNA (Figure 1D). A similar accumulation of viral RTCs in the cell periphery was observed upon transfection of anti-dynein antibodies prior to infection [7].

HIV-1 virions pseudotyped with VSV G are delivered through clathrin-mediated endocytosis [35] and are further released into the cytoplasm following acidification of endosomes [36]. Clathrin-mediated entry relies mainly on actin filaments [37], while maturation of endosomes involves microtubules [38] and possibly the dynein motor complex [32]. Therefore, in order to confirm that the effects observed in Figure 1B-D were not stemming from pseudotyping, we repeated the experiment using a virus harboring an autologous HIV-1 envelope. U373-derived MAGI cells [39] were infected with the replication-competent HIV-1_NL43_. MAGI cells were transfected with the DHC-targeting siRNA or with the control siRNA and later infected with HIV‑1_NL43_, or left uninfected (Figure 1E). We used a shorter duration for the infection, as these non-pseudotyped viruses fuse primarily at the surface of cells and do not need to escape endosomes. We observed a significant increase in the amount of CA foci per cell following DHC depletion (Figure 1F). Specifically, the average number of CA foci per cell was 16.1 ± 2.3 in cells (*n* = 77) transfected with DHC siRNA compared to 5.4 ± 0.4 in control cells (*n* = 84) (*p* = 0.0001). CA foci were predominantly found in the vicinity of the nucleus in control cells (Luc siRNA), with a median relative distance to the nucleus of 0.30 (95% confidence interval, 0.26–0.37; *n* = 29 cells analyzed) (Figure 1G). As previously, DHC depletion caused a significant (*p* < 0.0001) shift in the distribution of CA cores towards the cell periphery; CA foci were found at a median relative distance of 0.70 (95% confidence interval, 0.63–0.75; *n* = 12 cells analyzed). These observations confirm that the effects reported in Figure 1B–D did not result from pseudotyping, and suggest that the dynein motor complex might not only be involved in the intracellular transport of CA cores (as reflected by the shift in relative distance) but also in their uncoating (as evidenced by their increased numbers). Collectively, the results shown in Figure 1 suggest that dynein motor complexes influence both HIV-1 retrograde transport and CA core stability in infected cells, irrespective of the mode of viral entry.

### 2.2. DHC Depletion Alters HIV-1 Uncoating, As Analyzed Using the Fate-of-Capsid Assay

**Figure 2:**
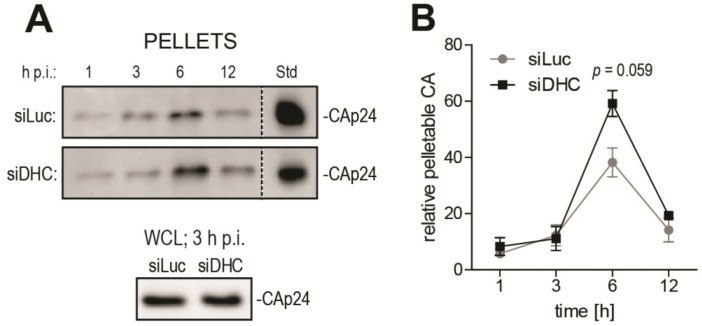
DHC depletion affects HIV-1 uncoating as analyzed using the fate-of-capsid assay. (**A**) Time-course fate-of-capsid assay. HeLa cells were transfected with siRNAs targeting DHC (siDHC) or luciferase (siLuc) and then infected with HIV-1_NL43-GFP_ for 1 h. Pelletable CA cores were isolated by ultracentrifugation at the indicated times after infection and analyzed by Western blotting (upper panels). Also shown is a Western blot analysis of CAp24 in whole cell lysates (WCLs) at 3 h p.i. (lower panel). Identical amounts of a control CAp24-containing sample were included in each gel as an internal standard (“Std”); (**B**) Bands corresponding to pelletable CAp24 were quantified by densitometry up to 12 h p.i. and plotted after normalization to the standard. Mean values from two independent experiments are shown, with standard errors of the mean (SEM).

As an independent, biochemical approach to analyze uncoating, we used the well described fate-of-capsid biochemical assay that allows isolation of post-entry CA cores from infected cells, by ultracentrifugation of pre-cleared cell lysates through a sucrose cushion [22,40]. This assay has been mostly used to study the destabilization of HIV-1 cores caused by TRIM5 proteins [22,33,41], but several reports established that it was a suitable tool to analyze uncoating in other contexts [24,42,43]. In order to gain insight into the timing of HIV-1 uncoating and how it is affected by DHC depletion, a time-course fate-of-capsid assay was performed (Figure 2). HeLa cells transfected with siRNAs targeting either DHC or Luc were infected with equal amounts of VSV G-pseudotyped HIV-1_NL43-GFP_ for a short period of time (1 h) and then lysed immediately or placed in virus-free medium and lysed at 3, 6, 12 and 24 h post infection (p.i.). This allowed us to analyze uncoating in quasi-synchronized conditions. To track relative changes in the amounts of particulate (pelletable) CA core, samples from different time points were analyzed side by side by CA Western blotting. The CAp24 content in whole cell lysates, as analyzed at 3 h p.i., confirmed equal input (Figure 2A). To enable comparison between control and DHC-depleted conditions, equal amounts of a CAp24-containing reference sample were loaded on the different gels (Figure 2A). The amounts of particulate CAp24 determined by densitometry were then normalized to the reference sample and plotted (Figure 2B). These amounts of particulate CA likely reflect a balance between the dynamics of CA core release from endosomes and its uncoating. In the control cells transfected with Luc siRNA, the relative amounts of particulate CA peaked at 6 h p.i. but were smaller at 12 h p.i. (Figure 2A,B), suggesting that uncoating had taken place for most RTCs by that time, as expected [8,9]. Increased levels of pelletable CA were observed at 6 h p.i. in cells transfected with DHC siRNA (Figure 2A,B). Specifically, DHC depletion increased CAp24 content in the pelletable fraction by ~1.5-fold (*P* = 0.059) relative to the control cells (Figure 2B). This is consistent with our previous report showing an increase in relative amounts of CA cores 6 h p.i. following DHC depletion or treatment with nocodazole and paclitaxel [33]. Interestingly, the increase in amounts of particulate CA caused by DHC depletion appeared to be transient, suggesting that uncoating was delayed rather than fully impaired.

### 2.3. Disruption of the Dynactin Complex Interferes with HIV-1 Uncoating

**Figure 3:**
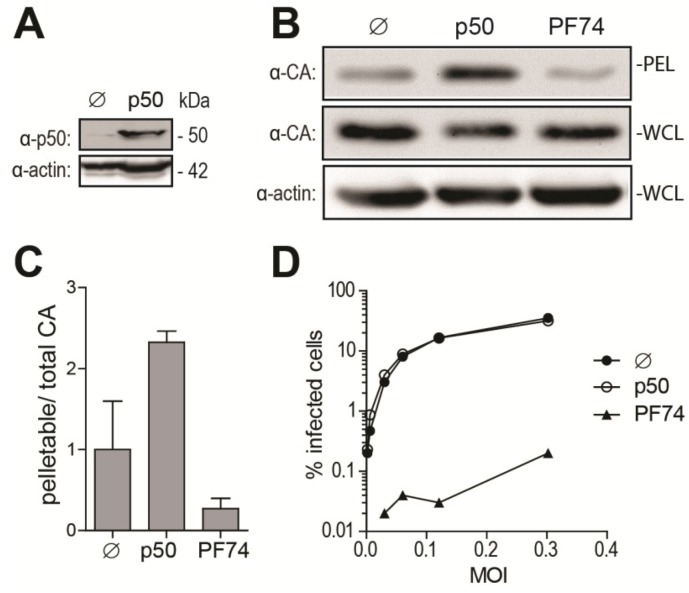
Over-expression of p50/dynamitin affects HIV-1 uncoating. (**A**) Western blot analysis of p50 and actin expression in HeLa cells 2 days after transfection with a plasmid encoding p50 or an irrelevant plasmid (pMIP; ø); (**B**,**C**) Fate-of-capsid analysis; (**B**) HeLa cells transfected with a p50 expression construct or with pMIP (ø), or treated with 10 μM of PF-3450074 (PF74), were infected two days later with HIV-1_NL43-GFP_. Supernatants were replaced with virus-free media, containing PF-3450074 where applicable, at 1 h p.i. and fate-of-capsid was performed at 6 h p.i. Shown are Western blot analyses of CAp24 in pellets and whole cell lysates (WCLs). Levels of actin in whole cell lysates were also analyzed; (**C**) CAp24 bands were quantified by densitometry in two independent experiments, and pelletable/whole cell lysate ratios were calculated and are shown as fold changes relative to the untreated control, with standard deviations; (**D**) Infectivity. HeLa cells treated as in (B) were infected with increasing amounts of HIV-1_NL43-GFP_. Supernatants were replaced with virus-free, drug-free medium 16 h p.i. The percentages of GFP-expressing cells were analyzed 2 days p.i. by flow cytometry.

As an additional approach to disrupt dynein-dependent transport, we over-expressed p50/dynamitin (p50), a subunit of the dynactin complex responsible for binding cargos to the dynein motor. p50 over-expression results in the disassembly of the dynactin complex, thereby disrupting cargo binding to the dynein motor and inhibiting dynein-dependent transport [44,45]. The efficiency of p50 transfection was confirmed by Western blotting (Figure 3A). We analyzed the effect of p50 over-expression on HIV-1 uncoating at 6 h p.i., since the maximal effect of DHC depletion on CA cores was observed at this time point (Figure 2). As a control, we used the small-molecule CA inhibitor PF-3450074 (PF74), known to disrupt HIV-1 infectivity through destabilization of the CA core [23]. p50 transfection resulted in increased amounts of pelletable CA cores isolated at 6 h p.i. from cells infected with HIV-1_NL43-GFP_ (Figure 3B). The pellet/whole cell lysate ratio was 2.3-fold (±0.097, *n* = 2) higher than for control cells transfected with an irrelevant plasmid (Figure 3C). As expected, PF-3450074 caused a decrease (3.7-fold) in the amounts of pelletable CA (Figure 3B,C). p50 transfection had no significant effect on HIV-1 infectivity, while PF-3450074 decreased permissiveness to HIV-1 by ~150- to ~550-fold, depending on the MOI used (Figure 3D) and as previously reported [46]. These results confirm that disruption of dynein-dependent transport counteracts uncoating, but the lack of an effect on infectivity suggests that uncoating is delayed rather than permanently impaired.

### 2.4. Dynein Depletion Reduces HIV-1 cDNA Levels

**Figure 4:**
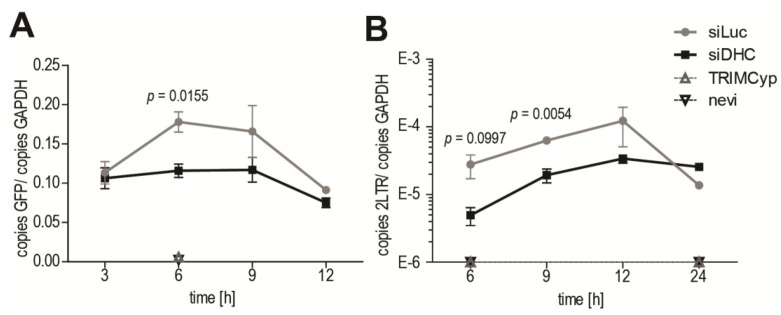
DHC depletion reduces levels of HIV-1 reverse transcribed DNA. (**A**,**B**) HeLa cells transfected with siRNAs targeting DHC (siDHC) or luciferase (siLuc), or treated with the RT inhibitor nevirapine (nevi; 20 µM), or stably expressing TRIMCyp (TCyp) were infected with HIV-1_NL43-GFP_. Supernatants were removed 3 h p.i. and replaced with virus-free media containing the appropriate drugs; Total DNAs were extracted at 3, 6, 9, 12 and 24 h p.i. and quantitative PCR (qPCR) was performed to detect GFP as a marker for RT (**A**), or 2-LTR circles as a marker for nuclear transport (**B**). GAPDH DNA was quantified for normalization purposes. The results are presented as ratios of GFP copies/GAPDH copies or 2-LTR copies/GAPDH copies. Shown are the means with SEM for each time point, from triplicate infections.

HIV-1 RT was suggested to be linked to uncoating of its CA core, as increased stabilization of CA cores was observed following inhibition of RT [8,15]. We therefore asked whether RT would be affected by treatments that have a stabilizing effect on CA cores. Quantitative PCR (qPCR) was performed on DNA isolated at various times from HeLa cells subjected or not to DHC depletion and infected with HIV‑1_NL43-GFP_. GFP DNA was quantified as a marker for late viral reverse transcribed DNA (Figure 4A). In control cells transfected with Luc siRNA, RT product levels peaked at 6 to 9 h p.i., which is consistent with previous reports [47,48,49]. In cells expressing owl monkey TRIMCyp, or treated with nevirapine, viral RT products were undetectable as expected [41] (Figure 4A). RT product levels in DHC-depleted cells at 6 h p.i. were 0.65-fold ± 0.027 those in the control cells, a small but significant decrease. However, no reduction was apparent at 12 h p.i., suggesting that the defect in RT was transient, consistent with the uncoating results in Figure 2. We also analyzed 2-LTR circular RT products, which are a marker for nuclear reverse transcribed HIV-1 DNA [50]. Less 2-LTR products were detected in DHC-depleted cells compared with the control cells at early time-points, but no difference was seen at 12 h p.i. (Figure 4B). Altogether, our results suggest that a transient decrease (or delay) in uncoating is associated with a transient RT defect.

## 3. Discussion

Previous articles showed that HIV-1 uncoating could be modulated by CA interactions with cellular factors such as CPSF6 [26] and cyclophilin A [24]. In this article, we present data suggesting that functional disruption of cytoplasmic dynein transiently increases the amounts of HIV-1 CA cores in cells, most likely reflecting delayed uncoating caused by inhibition of their transport towards the nucleus. These observations are in agreement with a recent report by the Campbell laboratory [43]. In this latter study, the authors similarly observed a delay in uncoating when dynein was targeted using siRNAs, as analyzed using a fate-of-capsid assay and also using an elegant “CsA withdrawal” assay that takes advantage of the fact that only intact CA cores can be inhibited by the restriction factor TRIMCyp in Owl monkey cells [8,51,52]. Our p50 over-expression experiment provides additional evidence for the involvement of dynein in HIV-1 uncoating. As observed using immunofluorescence microscopy, DHC depletion significantly increased the amounts of CA foci and resulted in their accumulation in the cell’s periphery. These results are consistent with the hypothesis that HIV-1 can use dynein motors to translocate on microtubules towards the nucleus [7]. Furthermore, fate-of-capsid experiments revealed that DHC depletion and disruption of dynein-dependent transport by p50 over-expression transiently increased the amounts of CA cores. Thus, dynein motor complexes seem to be involved in both HIV-1 trafficking and in its uncoating, supporting the idea that the two processes are related [13,53,54]. Along these lines, other authors previously reported that decreased uncoating resulting from mutations in CA correlated with decreased nuclear transport [55,56]. Consistently, mutations in CA affect the process of nuclear transport or the involvement of specific cellular factors in this process [56,57,58]. Binding of the HIV-1 CA protein, as part of intact CA cores, to the nucleopore protein NUP358 could trigger an uncoating step [59]. Collectively, these observations suggest that dynein complexes contribute indirectly to uncoating by transporting the HIV-1 CA core to the vicinity of nucleopores where key uncoating steps occur. Interestingly, a member of the PDZ domain-containing family, the putative microtubule-interacting protein PDZD8, was recently reported to stabilize HIV-1 CA cores [27], raising the possibility that HIV-1 uncoating is actively inhibited during transport. Whether dynein complexes are involved in this stabilization remains to be investigated.

Neither DHC depletion (not shown) nor p50 over-expression (Figure 3) had a significant effect on HIV-1 infectivity in HeLa cells, as we previously reported [33] and consistent with the fact that the effects of these interventions on uncoating and RT seem to be transient (Figure 2 and Figure 4). Although the effect of counteracting dynein on HIV-1 transport has long been known from live microscopy-based evidence [7], it was not clear whether inhibiting dynein completely disrupts HIV-1 transport, and our results suggest that it does not. In their recent paper, however, Lukic *et al.* [43] showed that pharmacological inhibition of cytoplasmic dynein by Ciliobrevin D does decrease infectivity. Why does this drug have an effect on infectivity while two genetic approaches (DHC knockdown and p50 over-expression) do not, is unclear, but one could propose off-target effects of the drug on infectivity, independent of its effect on dynein. It is equally possible, however, that our genetic interventions did not fully disrupt dynein function and that low levels of cytoplasmic dynein complexes are sufficient to achieve transport, although in a delayed fashion. Alternatively, it is possible that HIV-1 CA core retrograde transport can proceed in the absence of functional dynein, albeit less efficiently, by dynein-independent or even microtubule-independent mechanisms, such as translocation on actin microfilaments [60].

## 4. Materials and Methods

### 4.1. Cells, Pharmaceuticals and Antibodies

Human embryonic kidney 293T (HEK293T) cells, epithelial carcinoma HeLa cells and human U373-derived MAGI cells [39] were maintained in Dulbecco’s modified Eagle’s medium (DMEM) with high glucose, supplemented with 10% fetal bovine serum (FBS) and antibiotics at 37 °C, 5% CO_2_. HeLa cells retrovirally transduced to stably express owl monkey TRIMCyp were described before [41,61]. All cell culture reagents were from HyClone (Thermo Scientific, Logan, UT, USA). MG132 was from Sigma (St Louis, MI, USA), while PF-3450074 was provided by Pfizer (New York, NY, USA). Rabbit polyclonal antibodies against dynein heavy chain and p50/dynamitin were from Santa Cruz (Dallas, TX, USA) and Millipore (Billerica, MA, USA), respectively. Capsid (CA, p24) was detected using a mouse monoclonal antibody (clone 183, cat#3537) from the AIDS Research and Reference Reagent Program. The HRP-conjugated mouse anti-actin antibody was from Sigma. HRP-conjugated goat anti-rabbit and goat anti-mouse antibodies used as secondary antibodies in Western blots were from Santa Cruz.

### 4.2. Plasmid DNAs and Retrovirus Production

p50/dynamitin-HA was a gift from Tina Schroer [62]. To produce viral vectors, 10-cm culture dishes or 75-cm flasks of sub-confluent HEK293T cells were co-transfected using polyethylenimine (PEI; MW 25,000, Polyscience, Niles, IL, USA) with the appropriate plasmids, as follows: for the replication-competent HIV-1_NL43_, 20 μg of pNL4-3; for the viral vector HIV-1_CMV-GFP_, pTRIP-CMV-GFP (10 μg), pΔR8.9 (10 μg) and pMD-G (5 μg); for HIV-1_NL43-GFP_, pNL-GFP (10 μg) and pMD-G (5 μg) [63,64,65,66]. Media were changed 16 h post transfection and virus-containing supernatants were collected after an additional 1.5 days of culture. Viral stocks were clarified by centrifugation for 5 min at 400 × *g*.

### 4.3. Viral Challenges

Cells were seeded in 24-well plates at 2 × 10^5^ cells per well and challenged the next day with the appropriate GFP-expressing viral vectors. Where applicable, cells were pre-treated for 15 min with PF-3450074, and supernatants were replaced with fresh medium 16 h post infection (p.i.) Cells were trypsinized 48 h p.i. and fixed in 2% formaldehyde (Fisher Scientific, Waltham, MA, USA). The percentages of GFP-positive cells were then determined by analyzing 10^4^ to 3 × 10^4^ cells on a FC500 MPL cytometer (Beckman Coulter, Brea, CA, USA) using the CXP Software (Beckman Coulter). For p50 over-expression experiments, 10^6^ cells seeded in a 10-cm dish were PEI transfected with 5 μg of p50/dynamitin-HA or an irrelevant plasmid (pMIP). Cells were seeded in 24-well plates 24 h later and challenged with viral vectors the next day.

### 4.4. siRNA Transfection

For the siRNA treatments, 10^6^ cells were seeded in a 10-cm dish in Opti-MEM (Gibco, Carlsbad, CA, USA) and transfected the next day with 40 nM of siRNA using DharmaFECT 1 (Dharmacon, Lafayette, CO, USA). The siRNA targeting the sequence 5’GATCAAACATGACGGAATT of (DHC), has been described before [32,33] and was purchased from Qiagen (Venlo, The Netherlands). A control siRNA (5’CGTACGCGGAATACTTCGATT) targeting the luciferase mRNA [33] was purchased from Dharmacon. 48 h post transfection, cells were seeded in 24-well plates and infected the next day with HIV-1 vectors as described above.

### 4.5. Immunofluorescence Microscopy

HeLa and MAGI cells were siRNA-transfected as detailed above. 72 h later, 2 × 10^5^ cells were seeded on glass coverslips placed in 3.5-cm wells and were infected the next day at a multiplicity of infection (MOI) of ~1 with vesicular stomatitis virus glycoprotein (VSV G)-pseudotyped HIV-1_CMV-GFP_ (HeLa) or replication-competent HIV-1_NL43_ (MAGI) in the presence of 1 μg/mL MG132. Infections were carried out for 2 h (HIV-1_NL43_) or 4 h followed by a 2-h incubation in fresh medium (HIV‑1_CMV-GFP_). Cells were then fixed for 30 min in 4% formaldehyde-DMEM, followed by 3 washes with ice-cold phosphate buffer saline (PBS) and permeabilized for 2 min on ice in 0.1% Triton X-100, 0.1 mM sodium citrate. Cells were then washed again three times with PBS and treated with 10% normal goat serum (Sigma) containing 0.3 M glycine (Sigma) for 30 min at room temperature. This was followed by a 4-h incubation with antibodies against CA (1:100 dilution) in PBS containing 10% normal goat serum. Cells were washed five times and fluorescently stained with AlexaFluor488-conjugated goat anti-mouse (Molecular Probes, Eugene, OR, USA) diluted 1:200 in normal goat serum-containing PBS. Cells were then washed five times in PBS before mounting in Vectashield (Vector Laboratories, Peterborough, UK). Hoechst33342 (0.8 μg/mL; Molecular Probes) was added along with the penultimate PBS wash to reveal DNA. Z-stacks were acquired on an AxioObserver Microscope (Carl Zeiss Canada, Toronto, ON, USA) equipped with the ApoTome module, and the median optical slice of each Z-stack was analyzed. For the calculation of relative distances to the nucleus, we proceeded as described previously [33]. The nucleus edge was defined using Hoechst33342 staining, while the cell’s edge was visualized by bright field microscopy. The shortest distances to the nucleus edge and to the cell’s edge were calculated in 2D images of median optical slices and thus represent apparent, rather than real, shortest distances.

### 4.6. Fate-of-Capsid Assay

To analyze post-entry CA uncoating, a protocol adapted from Perron *et al.* [40] was used and has been described in details before [33,41]. 4 × 10^6^ HeLa cells, previously transfected with siRNAs or p50 as described above, were seeded in 10-cm dishes. The next day, if applicable, cells were pretreated 15 min with PF-3450074 and infected with HIV‑1_NL43-GFP_ at a MOI of ~2 and in presence or absence of PF-3450074. Supernatants were replaced with fresh media, containing PF-3450074 or not, 1 h p.i. Cells were collected by trypsinization at different time points, washed in ice-cold PBS and resuspended in 1.5 mL of ice-cold lysis buffer (100 μM Tris-HCl (pH 8.0), 0.4 mM KCl, 2 μM EDTA, Roche’s Complete protease inhibitor) and disrupted with a Dounce homogenizer. Whole cell lysate samples were collected at this point. Lysates were centrifuged for 5 min 1000 × *g*, 4 °C to remove cell debris and nuclei, and supernatants were layered on top of a 50% sucrose cushion prepared in STE buffer (100 mM NaCl, 10 mM Tris-HCl (pH 8.0), 1 mM EDTA). Particulate viral cores were sedimented by ultracentrifugation in a Sorval WX Ultra 100 ultracentrifuge at 175,000 × *g* for 2 h at 4 °C. Pellets were resuspended in 80 μL of 1X denaturing loading buffer and processed for CA Western blotting together with whole cell lysates.

### 4.7. Quantitative PCR of HIV-1 DNA

Cells were seeded in 12-well plates at 3 × 10^5^ cells/well and infected with HIV-1_NL43-GFP_ (MOI ~0.03 as calculated on the control permissive cells) depleted or not of DHC, as detailed above. Prior to infection, viral stocks were passed through 0.45-µm filters and pretreated for 1 h at 37 °C with 20 U/mL DNAse I (New England Biolabs) to prevent contamination by carry-over plasmid DNA. After 3 h, supernatants were replaced with fresh media, and cellular DNAs were isolated at various times using the DNeasy Blood and Tissue Kit (Qiagen, CA, USA). Purified DNAs were digested with 0.25 µL of DpnI (20 U/µL, New England Biolabs) for 1 h at 37 °C to help remove any remaining plasmid DNA. The absence of such contaminant DNA was verified by performing control infections in the presence of 80 µM of the RT inhibitor nevirapine. The primers and reaction conditions for detecting GFP, 2-LTR and GAPDH have been previously described [67]. In each experiment, a standard curve of the amplicon being measured was run in duplicates ranging from 30 to 3 × 10^5^ copies plus a no-template control. Reactions contained 1x SensiFAST SYBR Lo-Rox (Bioline, UK), 300 nM forward and reverse primers, and 100–300 ng template DNA. Results were analyzed with the MxPro software (Agilent Technologies, CA, USA). Computed values for GFP and 2-LTR copy numbers were normalized to the amounts of GAPDH copy numbers for each sample.

### 4.8. Statistical Analysis

All statistical analyses were done using GraphPad Prism [68]. The box blot representations in Figure 1 were generated using the R software [69].
